# The Role of Physical Examination and Clinical Scores in Distinguishing Streptococcal Colonization from Pharyngitis in Pediatric Patients: Insights from a Common Clinical Scenario

**DOI:** 10.3390/microorganisms13030529

**Published:** 2025-02-27

**Authors:** Victor Daniel Miron, Doina Anca Pleșca, Anuța Bilașco, Claudiu Filimon, Sigrid Covaci, Anca Cristina Drăgănescu

**Affiliations:** 1 Carol Davila University of Medicine and Pharmacy, 050474 Bucharest, Romania; victor.miron@umfcd.ro (V.D.M.);; 2 National Institute for Infectious Diseases “Prof. Dr. Matei Balș”, 021105 Bucharest, Romania; 3 Children’s Clinical Hospital Dr. Victor Gomoiu, 022102 Bucharest, Romania; 4 Emergency Clinical Hospital, 014461 Bucharest, Romania

**Keywords:** influenza, *Streptococcus pyogenes*, group A streptococcus, streptococcal pharyngitis, colonization

## Abstract

The accurate differentiation between asymptomatic carriage with group A streptococcus (GAS) and active streptococcal pharyngitis is a complex task with important clinical and public health implications. This work aims to highlight the key strategies necessary for optimizing the diagnostic and therapeutic management of pediatric pharyngitis. Clinical scores are essential tools for improving diagnostic accuracy. When combined with laboratory tests such as throat cultures and rapid antigen detection tests, these systems enable effective risk stratification of patients, supporting more precise treatment decisions. In addition to diagnostic strategies, the article underscores the importance of patient-centered communication, particularly with the families of pediatric patients. Clear, empathetic discussions about the condition, diagnostic rationale, and treatment plan help foster trust, enhance adherence to medical recommendations, and reduce anxiety related to potential complications. A critical outcome of these combined strategies is the reduction of unnecessary antibiotic use, which plays a pivotal role in preventing both overdiagnosis and overprescription. This, in turn, mitigates the growing threat of antimicrobial resistance, one of the most significant global health challenges. By integrating clinical expertise, standardized protocols, and effective communication, healthcare providers can promote judicious and effective management of streptococcal pharyngitis or asymptomatic carriage, contributing to improved individual and population health outcomes.

## 1. Introduction

*Streptococcus pyogenes* or group A streptococcus (GAS) is a bacterium commonly implicated in acute pathology in children. The clinical spectrum ranges from healthy carrier to invasive GAS (iGAS) infection. iGAS is characterized by the detection of GAS in sites of the body that are typically free of bacteria. This condition can present itself through various forms of symptoms such as bloodstream infections, streptococcal toxic shock syndrome, pneumonia with or without empyema, retropharyngeal abscess, osteomyelitis or septic arthritis, meningitis, or necrotizing fasciitis [1]. However, in children, the most prevalent clinical scenarios are attributable to localized forms of GAS infection, particularly acute streptococcal pharyngitis [2]. It has been documented that up to 30% of cases of sore throat in children are associated with streptococcal pharyngitis [3,4]. Nevertheless, the rate of antibiotic prescribing in children with sore throats exceeds two-thirds of cases [5], accounting for more than 5% of all antibiotic use worldwide [6].

GAS can typically colonize the oropharynx of healthy individuals. Rates of asymptomatic carriage exceed the incidences of streptococcal pharyngitis [7]. Thus, the likelihood of prescribing antibiotics to a child with upper respiratory tract infection (URTI) symptoms and GAS-positive throat culture or rapid antigen detection test (RADT) is high. In these cases, the clinical judgment of the physician is crucial. In this communication, we start with a case of a patient with a clinical scenario frequently encountered in daily clinical practice. The aim is to discuss the importance of clinical examination, the use of clinical diagnostic scores, and medical judgment in differentiating between asymptomatic GAS colonization and GAS pharyngitis, serving as a practical tool for daily clinical practice.

## 2. Clinical Scenario

A 2-year and 5-month-old male child was brought in January to the hospital by his parents with a history of high fever (maximum 39.8 °C), productive cough, and nasal congestion, which had started 30 h before the evaluation. The child had no chronic diseases and no history of hospitalization. He had received all recommended vaccinations in accordance with the National Immunization Program in Romania [8] but had not received any optional vaccines, including the influenza vaccine. He had been attending a childcare facility for approximately six months. The mother reported that multiple children in the kindergarten group exhibited fever and respiratory symptoms, and three of them were diagnosed with acute streptococcal pharyngitis by general practitioners or pediatricians and treated with antibiotics.

The clinical examination at presentation indicated a good general condition, with a temperature of 38 °C. No cutaneous rash or palpable lymphadenopathy was observed. A comprehensive respiratory assessment revealed a frequent productive cough and marked nasal congestion accompanied by rhinorrhea, but no signs of dyspnea or abnormal pulmonary auscultatory findings. Oropharyngeal examination demonstrated mild mucosal and tonsillar hyperemia without evidence of purulent exudates. No additional abnormalities were identified upon further physical examination.

Following a detailed review of the historical and clinical data, as well as considering the local epidemiological context of high influenza virus circulation, the decision was taken to perform a RADT for influenza and SARS-CoV-2. The RADT was positive for influenza A and negative for both SARS-CoV-2 and influenza B. Consequently, an RT-PCR for influenza viruses was conducted, and the result, obtained five hours later, confirmed an infection with influenza A(H1N1)pdm. Consequently, the decision was made to initiate antiviral treatment with oseltamivir.

A throat culture was performed at the mother’s insistence, as she was genuinely concerned about the reported cases in the kindergarten. However, considering the child’s age, medical history, symptoms, clinical examination findings, and the high circulation of influenza in the local epidemiological context, the established treatment (comprising antiviral and symptomatic drugs) was not modified, as the mother was informed about the low likelihood of GAS pharyngeal infection. Modified Centor (McIsaac) score [9] and FeverPAIN score [10] were consequently calculated and interpreted, and the results were explained to the mother. The results of these scores indicated a probability of 2 points, thus indicating a low likelihood of streptococcal infection (Table 1).

Four days after the initial presentation, the child returned to the hospital for re-evaluation. At that time, it was noted that the child had become afebrile shortly after the initiation of antiviral therapy, with a marked improvement in overall condition. Clinical examination revealed no abnormalities. However, a throat swab culture obtained at the previous visit was positive for GAS. Given the favorable clinical evolution and the low-risk profile indicated by clinical findings and the initially calculated clinical scores, it was concluded that the child was likely a healthy carrier of GAS. Therefore, no additional therapeutic measures or further monitoring were deemed necessary.

## 3. Discussion

In this communication, we aim to highlight the importance of clinical examination the use of clinical scores, and the integration of laboratory tests within the broader clinical context to accurately differentiate between GAS healthy carriers and streptococcal pharyngitis. The antimicrobial resistance rate of GAS is on the rise, especially to macrolides and clindamycin [11,12,13,14], although cases of resistance have also been reported for fluoroquinolones [15,16], penicillins [17], ceftriaxone [18], and even vancomycin [18,19,20]. This is worrying because many of the antibiotic prescriptions worldwide are for sore throat or acute pharyngitis [21,22]. However, although GAS is the main pathogen associated with acute pharyngitis in children, viruses are much more commonly involved in the inflammation of the pharynx and tonsils and sore throat. Therefore, in the majority of cases of acute pharyngitis in children, antibiotic treatment is not necessary, and the illness is self-limited. In differentiating asymptomatic colonization from streptococcal pharyngitis, a careful examination of the patient is required, together with medical judgment based on the patient’s history, changes noted on clinical examination, and the use of clinical scores developed over time to make a positive diagnosis of GAS pharyngitis. The identification of GAS by RADT or throat culture does not justify antibiotic administration. In our discussion, we will briefly review the epidemiology and clinical features of acute streptococcal pharyngitis, the prevalence and mechanisms of asymptomatic GAS carriage, and emphasize the importance of sound medical judgment in distinguishing between asymptomatic GAS colonization and acute GAS pharyngitis.

### 3.1. Streptococcal Pharyngitis: Epidemiological and Clinical Data

Almost all children will have at least one episode of GAS pharyngitis by adulthood. The majority of cases occur in children aged 5–15 years, particularly during time spent in the community [23]. Cases of acute GAS pharyngitis are rare in children under 3 years of age, although cases have been described in infants [24,25].

In a recent meta-analysis, Miller et al. [3] calculated a global incidence of GAS sore throat of 22.1% per year, but there is heterogeneity in the distribution of cases by geographic region, with rates of up to 40% reported in Saudi Arabia [26], 37% in the United States [27], 29.9% in Romania [4], 26.2% in Canada [28], 26.1% in South Africa [29], 21.4% in France [30], 14.3% in Turkey [31], 12% in Brazil [32], 10.7% in Ethiopia [20], 8% in Thailand [33], 8% in Australia [34], and 4.4% in Japan [35]. These data are approximate because even within the same country, the values can be different depending on the pediatric group analyzed and the population structure analyzed. In addition, GAS pharyngitis cases vary seasonally; thus, an increase in the number of cases has been observed in winter, when children spend more time indoors in collective settings [36]. Hervas et al. [37] showed that GAS activity increases with decreasing mean temperature and increasing solar radiation. However, peaks of cases have been described even during the warm season, in the months of June–July [4].

The clinical picture of uncomplicated streptococcal acute pharyngitis in children is dominated by sore throat and fever. This may be accompanied by difficulty swallowing, headache, abdominal pain, vomiting, decreased appetite, and malaise. Adolescents may often present with only severe sore throat with difficulty swallowing without fever as the only symptoms of GAS pharyngitis. Clinical examination may often reveal hyperemia of the oro-pharyngeal mucosa and uvula, red spots on the soft palate, and a furry tongue [21]. Laterocervical and submandibular adenopathies are also often present, without inflammatory signs [38]. For infants and toddlers, typical clinical manifestations are not as frequent. Commonly, they have a subacute presentation with low-grade fever, agitation, decreased appetite, nasal congestion, and rhinorrhea [39,40]. In contrast, the presence of nasal congestion, rhinorrhea, cough, and conjunctivitis significantly decreases the likelihood of streptococcal etiology in acute pharyngitis in children over three years of age [21,41]. It is obvious that each child may have its own unique response to GAS pharyngeal infection; therefore, clinical suspicion should be based on signs, symptoms, epidemiologic context, and a complete and thorough history of the disease.

The positive diagnosis is established on clinical signs and symptoms, based on the clinical scores mentioned above (modified Centor score and FeverPAIN score) and by the presence of GAS in the oropharynx. Throat culture is still considered the gold standard [41], however, it has the disadvantage of long processing time until a result is confirmed; therefore, in practice, RADT is preferred, with quick results in just a few minutes. RADT, however, is subject to a higher likelihood of false negative or false positive results. An incorrect collection technique or sampling shortly after the child has eaten or drunk may give false-negative results, while false-positive results may occur due to the persistence of streptococcal antigens in the oropharynx, even in the absence of viable GAS strains [21,42]. In addition to throat culture and RADT, nucleic acid amplification tests (NAAT) can also be used. These have a higher sensitivity than RADT and provide results in a shorter time than culture. The main disadvantage for clinical practice is the higher cost of NAAT compared to both RADT and culture. None of the three types of tests can differentiate between active infection and colonization. It is therefore essential to correctly select patients requiring testing to prevent misdiagnosis and unnecessary antibiotic administration. Because we have previously noted the low incidence of GAS pharyngitis in children under 3 years of age, it is important to note that the Infectious Diseases Society of America recommendation is to not routinely test for GAS in children under 3 years of age or children who, regardless of age, have epidemiologic and clinical features suggestive of a viral etiology of acute pharyngitis [41]. Also, asymptomatic contact testing or post-treatment testing is not recommended as a routine practice. It is intended for special situations, outbreaks, patients at high risk of post-streptococcal complications, or patients with recurrence of symptoms suggestive of acute GAS pharyngitis [41].

Regarding the antimicrobial treatment of GAS pharyngitis, several studies have demonstrated the benefits of reducing the treatment duration from 10 days to 6 [43] or even 5 days [44] without increasing the risk of post-streptococcal complications. A consensus on GAS pharyngitis treatment is needed to facilitate an update of treatment protocols that would be globally accepted.

### 3.2. Asymptomatic GAS Carriage: Prevalence, Mechanisms, and Impact

Asymptomatic GAS carriage in the school-age pediatric population is a frequent and dynamic event. In a four-year follow-up study of school-aged children, 53% of them were at least once identified as healthy GAS carriers, and the overall prevalence of GAS colonization during the study was 48% [45]. In 2010, a meta-analysis by Shaikh et al. showed a prevalence of GAS colonization of 12% among healthy children aged 5–17 years [27]. After more than 15 years, it appears that the prevalence of GAS among healthy carriers has remained at similar levels in the vast majority of countries. Thus, values of 10.1–10.8% have been reported in Nepal [46,47], 12.8% in Yemen [48], 10% in the United Arab Emirates [49], 13.9% in Indonesia [50], 14.3% in Turkey [31], 8.3% in Australia [51], 9.5% in Kenya [52], 11.5% in Pakistan [53], 10.6–12.2% in Ethiopia [54,55,56], and slightly higher rates in Argentina (14.2%) [57] or Uganda (15.9% [58], and the United States (up to 20%) [59]. As in the case of pharyngitis, the incidence of cases can vary even within the same community, depending on the season, population density, size of the study group, and the testing method used. Tanz et al. showed that the probability of finding GAS by molecular methods is significantly higher, 20.3% compared to 12.5% for bacterial throat culture [60]. Age seems to be another relevant factor, directly correlated with GAS colonization rates, as they are inversely proportional to age [61]. However, reports have been published showing that social and environmental factors can lead to high rates of GAS colonization even among older children and adolescents [57]. Some studies have shown a higher prevalence of GAS colonization among girls [46,47,48,55,56].

Seasonality is another important factor in modulating GAS colonization rates, so rates appear to be higher in the cold season compared to the warm season [62]. GAS colonization also varies according to the environment of residence, but the data are not always consistent. Most studies show a higher prevalence of colonization in urban compared to rural areas [55,56,58]. However, research conducted strictly in rural areas shows significantly increased rates of colonization [57]. An important correlation has been identified between the population density in the home environment, kindergartens, or schools and the rate of GAS colonization, which highlights the impact of crowding on the spread of the bacteria [46,54,55].

In addition to these factors, further studies have attempted to identify other determinants of colonization. Children from low-income families are at increased risk of colonization, most likely due to poor hygiene, inadequate nutrition, and poor living conditions [50,55,56,63]. A history of hospitalization is also associated with a higher likelihood of colonization compared to children who have not been hospitalized [55,56].

Another significant factor is tonsillar hypertrophy, identified as a positive predictor for asymptomatic GAS carrier status. Rahmadhany et al. [50] demonstrated this in their study of a group of children from low socioeconomic status families living in crowded households. In addition, exposure to cigarette smoke appears to increase the likelihood of GAS carriage, highlighting the role of environmental factors in this process [56].

The oro-pharyngeal asymptomatic GAS carriage is influenced by many factors, some of which are host-dependent, while others are related to the bacteria, especially virulence factors. The commensal bacterial flora of the oral mucosa, the amount of salivary immunoglobulins, and their specificity have an important impact on colonization [64]. Given the high variability of the M protein in the GAS structure, it is assumed that specific immunity does not prevent GAS colonization or repeated episodes of GAS pharyngitis [65]. It is known that there is a significant genetic diversity of GAS strains. Genotyping of these strains involves sequencing the 5′ end of the *emm* gene. The *emm* gene encodes the M protein, which is a major virulence factor. To date, more than 250 *emm* types have been identified that determine specific virulence factors [66]. In a longitudinal study, Martin et al. failed to demonstrate an association between the type of *emm* and the likelihood of becoming a GAS carrier, but they found that healthy GAS carriers changed their *emm* type during the course of the study [67]. There are studies suggesting that certain types of *emm* are capable of producing biofilm (especially *emm6*), thus evading the host immune response and escaping the direct action of antibiotics, thus setting up colonization whose clearance by host response or treatment will be difficult to eradicate [68]. Also, Mengeloglu et al. found no significant differences in the type of *emm* between healthy carriers and patients with documented infection [69]. In addition, several studies have shown that colonized children are more likely to have GAS strains containing PrtF genes (PrtF1 or PrtF2), which are considered major virulence factors [70,71,72,73]. It is known that more than half of people who have oropharyngeal GAS colonization, despite not receiving antibiotic treatment, will have spontaneous clearance over several weeks [74,75].

Transmission of GAS from an asymptomatic carrier child to another child is typically limited by several factors. Colonized patients are thought to have a lower density of GAS bacteria in the oropharynx compared to those with an acute infection, which reduces the ability of transmission [76]. Moreover, the absence of respiratory symptoms, and therefore of nasal or pharyngeal secretions, helps to reduce the risk of spreading the bacteria [77]. Davies et al. showed that GAS can lose its virulence over time, becoming less able to cause infection [78]. Also, asymptomatic colonized children are not considered at significant risk of developing complications associated with GAS infections. However, in developing countries, where acute rheumatic fever is endemic, colonization may represent a public health problem [79].

GAS colonization often confuses the clinical evaluation of patients with symptoms of acute pharyngitis because a positive GAS test result may be coincidental in the case of an underlying viral infection. This may lead to misdiagnosis and unnecessary prescription of antibiotics. Furthermore, no association has been established between asymptomatic GAS colonization and recurrent episodes of GAS infections [57].

Managing children colonized with GAS is challenging. In most situations, antibiotic treatment is not necessary and should not be recommended, according to guidelines developed by the Infectious Disease Society of America [41]. According to the Committee on Infectious Diseases of the American Academy of Pediatrics, there are indicated exceptions in which antibiotic treatment may be considered in healthy GAS carriers. These include (1) a family history of rheumatic fever or rheumatic heart disease to prevent the risk of severe complications in patients with predisposing factors; (2) family anxiety about GAS colonization (“streptophobia”), especially in cases where tonsillectomy is planned to eradicate colonization; (3) community outbreaks of streptococcal pharyngitis or scarlet fever to limit the spread of infection among susceptible individuals; and (4) eradication of “ping-pong” in cases of recurrent streptococcal pharyngitis, where repeated transmission among family members or other close contacts necessitates intervention for infection control [80].

### 3.3. Clinician’s Judgment in Differentiating Between Asymptomatic GAS Carriage and GAS Pharyngitis

The differentiation between asymptomatic *Streptococcus pyogenes* carriage and an active infection is essential in clinical practice to prevent overdiagnosis and unnecessary antibiotic treatment. This judgment requires careful integration of clinical, epidemiological, and laboratory data. Correct assessment involves identifying a fine balance between signs and symptoms suggestive of acute GAS pharyngitis, diagnostic test results (RADT, NAAT, or throat culture), and knowledge of the local prevalence of colonization. Clinical scores such as modified Centor and FeverPAIN are useful tools in guiding clinical decision-making. Studies have shown that these scores can significantly reduce unnecessary antibiotic prescribing by risk stratification of streptococcal pharyngitis. A low score suggests a low probability of streptococcal infection and supports a watchful waiting approach [21]. For example, in the clinical case discussed above, the interpretation of the clinical scores showed that the patient had a low probability of streptococcal pharyngitis, which guided the decision not to initiate antibiotic treatment. However, clinical scores should be used with caution in children under three years of age, where typical manifestations of streptococcal pharyngitis are rare, and existing tools have lower validity [41].

A positive GAS result, regardless of the test method used, does not confirm streptococcal etiology of an acute URTI. Similarly, a negative result cannot completely exclude streptococcal etiology. It is therefore essential that the clinician integrate all available information into the decision-making process. Clinical judgment involves correlating the laboratory results with the detailed history of the illness episode, including the onset, duration, and course of symptoms. It is also important to obtain information about the epidemiological context, such as similar cases in communities or local outbreaks of streptococcal pharyngitis. Unfortunately, in practice, this process is often influenced by factors such as high workload, time pressure, and lack of resources, particularly in emergency departments. Thus, some physicians fail to allow sufficient time to perform a complete and detailed clinical examination or to note all the changes identified. This can lead to incomplete diagnosis or overdiagnosis. Expanded clinical experience and ongoing training can contribute significantly to optimizing the diagnostic and treatment process. It is very important in differentiating infection and colonization, in addition to the clinical scores detailed above, to emphasize the presence or absence of upper respiratory symptoms such as nasal congestion, rhinorrhea, cough, conjunctivitis, and mouth thrush. These are frequently associated with a viral etiology, and their presence reduces the likelihood that the acute URTI is caused by GAS [41]. The epidemiologic context can significantly influence clinical judgment. For example, during periods with an increased prevalence of viral infections, the likelihood of a positive result reflecting colonization is higher. Also, factors such as community outbreaks of streptococcal pharyngitis and history of close contact with infected persons may influence therapeutic decisions.

The use of blood tests, especially white blood count, neutrophil count, and biomarkers such as C-reactive protein and procalcitonin, has been explored as a means to differentiate between bacterial and viral infections, particularly in cases where clinical presentation is inconclusive. More recently, myxovirus resistance protein A (MxA) [81,82,83,84] has gained attention as a promising viral infection marker, especially when used in combination with CRP, offering improved specificity in distinguishing bacterial from viral etiologies. While these blood tests and biomarkers can provide valuable insights, their routine use in pediatric practice remains controversial [85,86,87]. In outpatient settings, where most cases of acute pharyngitis are managed, access to blood tests is often limited, and the need for invasive investigations should be carefully weighed against their clinical utility. Moreover, given the high prevalence of viral infections in young children and the significant rates of asymptomatic GAS carriage, unnecessary testing may contribute to increased parental anxiety and lead to overuse of antibiotics if results are misinterpreted. Therefore, while these biomarkers may be useful in selected cases where there is significant diagnostic uncertainty, clinical judgment remains paramount in guiding management decisions.

It is crucial to recognize that asymptomatic carriage of GAS in the oro-pharynx carries the potential to progress into invasive infection under certain conditions. Consequently, children presenting with clinical features indicative of viral pharyngitis, who nonetheless test positive for GAS, warrant careful monitoring. This approach is essential to promptly identify any signs of disease progression. Parents and caregivers should be thoroughly educated on the warning signs that necessitate a prompt return for medical evaluation. These include the persistence of high fever, worsening pharyngeal pain associated with difficulty in swallowing, and a decline in the child’s general condition symptoms, which may signify the activation or transition of GAS from a commensal to a pathogenic state. In light of this, non-antibiotic interventions aimed at preventing disease escalation may prove beneficial. For instance, the use of topical antiseptics containing methylene blue or chlorhexidine can help reduce local microbial load, thereby mitigating the risk of GAS overgrowth and subsequent invasion. Furthermore, rebalancing the oral microbiota through strategies such as probiotics or oral care optimization could play a preventive role by enhancing mucosal immunity and competitive inhibition of pathogenic strains. These measures, in combination with vigilant observation, offer a pragmatic alternative to the immediate prescription of antibiotics, which carries the risk of promoting antimicrobial resistance and disturbing the balance of the commensal flora.

An essential component in the management of acute pharyngitis is family education and information. Providing clear and comprehensive explanations regarding the self-limiting nature of viral pharyngitis, alongside the risks associated with unnecessary antibiotic use, plays a pivotal role in mitigating anxiety and fostering adherence to medical recommendations. Educating families about potential complications and the importance of symptom monitoring empowers them to actively participate in the care process and reduces the likelihood of premature or inappropriate antibiotic requests. The use of evidence-based educational tools, such as informational brochures, interactive mobile applications, or video tutorials, has been shown to enhance knowledge retention and engagement. These resources can help reinforce key messages about symptom recognition, the expected course of illness, and when to seek further medical attention. Studies indicate that well-informed patients and caregivers demonstrate higher levels of trust in healthcare providers, leading to improved compliance with prescribed treatment plans and a reduction in unnecessary healthcare visits. Furthermore, tailored education can reduce antibiotic overprescription rates, contributing to global efforts to combat antimicrobial resistance [36,88,89,90]. Incorporating such educational strategies within routine pediatric care enhances the therapeutic alliance between healthcare professionals and families.

## 4. Conclusions

Accurate differentiation between asymptomatic GAS colonization and streptococcal pharyngitis is a multifaceted process that requires clinicians to integrate clinical presentation, laboratory findings, and epidemiological context. This diagnostic challenge emphasizes the importance of clinical scoring systems, which provide an evidence-based framework to guide decision-making. When used in conjunction with laboratory tests, these clinical scores improve diagnostic accuracy and help stratify patients by risk. Equally critical is effective communication with the patient’s family, particularly regarding the nature of the condition, the rationale behind diagnostic and therapeutic choices, and the importance of symptom monitoring. Additionally, by aligning family expectations with clinical evidence, the likelihood of unnecessary antibiotic use can be significantly reduced. The implementation of these principles plays a crucial role in minimizing overdiagnosis, preventing antibiotic overprescription, and thereby reducing the risk of antimicrobial resistance. Through a combination of clinical expertise, evidence-based protocols, and patient-centered communication, healthcare providers can ensure more judicious and effective management of pediatric pharyngitis, promoting both individual and public health outcomes.

## Figures and Tables

**Table 1:** Modified Centor (McIsaac) score [9] and FeverPain score [10] results.

Modified Centor Score		Our Case	FeverPAIN Score		Our Case
Age 3–14 years	1 point	No—0 point	Fever in past 24 h	1 point	Yes—1 point
Presence of exudate or swelling on tonsils	1 point	Yes—1 point	Absence of cough or coryza	1 point	No—0 point
Presence of ender/swollen anterior cervical lymph nodes	1 point	No—0 point	Symptom onset ≤ 3 days	1 point	Yes—1 point
Temperature ≥ 38 °C	1 point	Yes—1 point	Purulent tonsils	1 point	No—0 point
Absence of cough	1 point	No—0 point	Severe tonsil inflammation	1 point	No—0 point
Total		2 points	Total		2 points

## Data Availability

The original contributions presented in the study are included in the article, further inquiries can be directed to the corresponding author.
